# A Comparison of the Efficacy and Safety of Ustekinumab and Upadacitinib in Biologically Experienced Ulcerative Colitis Patients

**DOI:** 10.3390/biomedicines13102455

**Published:** 2025-10-09

**Authors:** Osman Özdoğan, Serkan Yaraş, Mehmet Kasım Aydın, Fehmi Ateş, Engin Altıntaş, Orhan Sezgin

**Affiliations:** Department of Internal Medicine (Gastroenterology), Faculty of Medicine, Mersin University, 33343 Mersin, Turkey; drserkan1975@hotmail.com (S.Y.); mkasim.aydin3321@gmail.com (M.K.A.); drfehmiates@hotmail.com (F.A.); enginaltintas@gmail.com (E.A.); drorhansezgin@gmail.com (O.S.)

**Keywords:** ustekinumab, upadacitinib, ulcerative colitis, efficacy, safety, biomarkers, real-world evidence

## Abstract

**Background/Objectives:** Ustekinumab (UST) and upadacitinib (UPA) are molecules that have been used in patients with ulcerative colitis (UC) since 2019 and 2022, respectively. Both agents are generally preferred for biologically experienced UC patients. However, the number of head-to-head studies comparing the efficacy and adverse events of UST and UPA in this patient group is limited. **Methods:** This was a retrospective cohort study evaluating the efficacy and safety of UST (*n* = 57) and UPA (*n* = 32) in biologically experienced UC patients during the induction and 24-week maintenance treatment periods. Most patients in both groups had received prior anti-TNF treatment (98.2% and 96.9%, respectively). Clinical response and remission rates were determined based on the partial Mayo score (PMS). Additionally, patients’ pre-treatment laboratory parameters were compared with their results at week 24. **Results:** During the induction phase, clinical response and remission were achieved in 84.2% and 43.9% of the UST group and 93.8% and 50% of the UPA group, respectively (OR [95% CI] = 2.81 [0.57–6.87] and 1.28 [0.54–3.05]). At week 24, the clinical response and remission rates in the UST and UPA groups were similar (77.1% vs. 80% and 58.3% vs. 63.3%, respectively). No statistically significant difference was found between the groups (*p* > 0.05). Both UST and UPA provided a marked reduction in fecal calprotectin and CRP levels. Regarding safety, UPA treatment led to increased total, LDL, and HDL cholesterol levels, whereas UST did not. In both groups, glucose; HbA1c; and thyroid, renal, and liver functions remained stable. No serious adverse events were observed in either group. At week 24, treatment continuation rates were 68.4% (*n* = 39) for UST and 78.2% (*n* = 25) for UPA (OR = 0.61 [0.22–1.66]). **Conclusions:** In biologically experienced ulcerative colitis, both UST and UPA are effective and safe treatment options. This study did not statistically demonstrate the superiority of UPA over UST. Given the preliminary nature and limited patient numbers of this investigation, our findings require confirmation through future multicenter, large-scale, and long-term prospective studies.

## 1. Introduction

Ulcerative colitis (UC) is a chronic, idiopathic inflammatory disease characterized by unpredictable periods of flares and remission [1]. In patients with moderate to severe UC who do not respond to conventional therapies such as aminosalicylates and immunomodulators (azathioprine, 6-mercaptopurine, methotrexate), new therapeutic molecules have been developed over the last two decades. These molecules include biological agents, such as tumor necrosis factor (TNF)-α antagonists, anti-integrin agents, and interleukin (IL)-12/23 inhibitors, as well as small-molecule drugs like Janus kinase (JAK) inhibitors and sphingosine 1-phosphate receptor modulators [2,3].

Ustekinumab (UST) is a fully human IgG1κ monoclonal antibody that binds to the shared p40 subunit of interleukin-12 (IL-12) and interleukin-23 (IL-23) cytokines. This action prevents the activation of the Th1 and Th17 cytokine pathways, which play a central role in the pathogenesis of UC [4]. Following the UNIFI phase study published in 2019, UST was approved for use in patients with moderate to severe active UC who had an inadequate response, loss of response, or intolerance to conventional therapies or other biological agents [5]. The 2024 American Gastroenterological Association (AGA) living guideline classifies UST as an agent with moderate efficacy in bio-naïve UC patients; however, it evaluates it among drugs with high efficacy, particularly in anti-TNF-experienced patients [6]. The UNIFI study reported an 8.5-point difference in the clinical remission rate in the induction phase for bio-naïve patients compared with placebo (18.4% vs. 9.9%), with this difference increasing to 11.5 points (12.7% vs. 1.2%) for patients with prior biological exposure [7]. Another study also stated that UST is a less favored option in bio-naïve patients compared with anti-TNF agents and vedolizumab [8]. Real-world data on UST use also entirely come from biologically experienced patients [9,10,11]. In some countries, such as Turkey and Finland, the use of a biological agent like anti-TNF before UST is a prerequisite for reimbursement [12].

Janus kinase (JAK) inhibitors block the intracellular phosphorylation and subsequent activation of key cytokine signaling pathways involved in inflammatory diseases. Upadacitinib (UPA) is an oral small-molecule drug that more potently inhibits JAK1 compared with other JAK isoforms (JAK2, JAK3, TYK2 [tyrosine kinase]) [13]. The U-ACHIEVE and U-ACCOMPLISH phase studies have demonstrated the high efficacy of UPA in UC patients [14]. Subsequently published studies and network meta-analyses have shown that UPA is one of the most effective and leading agents used in UC treatment [15,16,17]. UPA has been approved by both the U.S. Food and Drug Administration (FDA) and the European Medicines Agency (EMA) for the treatment of moderate to severe active UC. However, due to risks such as serious infections, malignancies, major adverse cardiovascular events (MACEs), and thrombosis reported in previous studies of this drug class (particularly the ORAL Surveillance study on Tofacitinib), the FDA recommends that JAK inhibitors be used in patients who have failed anti-TNF antagonists. The EMA advises caution when using them as a first-line treatment in patients aged 65 or older, smokers, or those with cardiovascular risk factors [6,18]. In Turkey, reimbursement for UPA necessitates prior failure with anti-TNF or vedolizumab therapy.

The main advanced molecular therapies used within the reimbursement system in Turkey are anti-TNF agents (infliximab, adalimumab), anti-integrin (vedolizumab), anti-interleukin (IL)-12/23 (ustekinumab), and upadacitinib. Of these molecules, UST has been in clinical use for over five years, while UPA has been in use for over one year.

As mentioned previously, UST and UPA are widely preferred for treating biologically experienced patients with moderate to severe ulcerative colitis. A comparison of real-world efficacy and safety outcomes between these two agents, which possess different mechanisms of action, is currently limited by the lack of available data. Ascertaining whether there is a difference in efficacy and analyzing the safety profiles of both treatments, including their effects on hematological parameters, liver and renal function tests, and other tests, are the goals of this study.

## 2. Materials and Methods

### 2.1. Study Design and Participants

The differences between UST and UPA regarding efficacy, side effects, and laboratory parameters were examined in this retrospective cohort preliminary study in patients with moderate to severe UC who had previously received biological treatment.

Patients included in this retrospective study were 18 years of age or older, had a prior loss of response to biological agents, had continued UST or UPA treatment for at least 6 months, and had regular follow-up data (sufficient clinical, laboratory, and/or endoscopic data at the beginning of treatment and during follow-up). Patients were excluded from the study (exclusion criteria) due to the following:

- Indeterminate colitis, isolated proctitis, fulminant colitis, toxic megacolon, microscopic colitis, or clinical findings suggestive of Crohn’s disease.
- Obstructive intestinal stricture, active ostomy, or a history of colectomy.
- Concurrent initiation of another new biological or small-molecule therapy not included in the study.
- A history of cardiovascular events (stroke, pulmonary embolism, deep vein thrombosis).
- The presence of chronic infections such as Hepatitis B or C or HIV, or active infections such as pneumonia or abscess.
- The presence of severe comorbidities such as liver cirrhosis or renal failure with a GFR value below 30 mL/min/1.73 m^2^.
- Pregnancy or breastfeeding.
- Initiation of a new drug or significant modification of an existing treatment regimen during the study period that could potentially affect lipid, glucose, or other laboratory values.

Azathioprine and other immunomodulator treatments were discontinued before UPA treatment initiation, as recommended. For patients on UST, if they were already on an immunomodulator, it was continued; if not, it was not started. Aminosalicylate doses were maintained at the same level as before. Some patients starting UST or UPA also received concomitant steroid therapy. Additionally, some patients required steroids during the maintenance phase due to clinical worsening. In our clinic, steroids were typically administered as 40 mg of methylprednisolone or its equivalent for 15 days, followed by a gradual taper over a period of at least 4 weeks and a maximum of 8 weeks, depending on the patient’s clinical status. The number of patients who were started on steroids is detailed in the Results Section.

The study was conducted within a gastroenterology unit that includes a specialized outpatient clinic dedicated solely to inflammatory bowel diseases. In this clinic, patient follow-up is guided by standard protocols. All patients were regularly evaluated at specified intervals, in addition to the induction and week 24 assessments. At each visit, treatment efficacy and the development of adverse events were recorded, activity scores were calculated, and laboratory values were collected according to routine procedures (e.g., after a minimum 8 h fast). As part of the routine visits, the condition of the colon was also examined via abdominal ultrasonography. Despite its retrospective nature, the study was meticulously conducted by excluding patients whose characteristics could compromise data quality or introduce bias.

The UST induction dose was administered as a single intravenous (IV) infusion based on weight (≤55 kg: 260 mg; >55 kg to ≤85 kg: 390 mg; and >85 kg: 520 mg). For maintenance therapy, a subcutaneous (SC) dose of 90 mg was administered every 8 weeks. While the initial evaluation of the induction response to UST in UC is recommended at week 8, current research and clinical phase studies suggest that patients with an inadequate response at this time may receive an additional SC dose, thereby extending the induction evaluation to week 16. This is noted to be acceptable due to the delayed onset of action of UST [5,19]. For this reason, we calculated the UST induction response at week 14. For UPA, the 45 mg induction dose was given for the first 2 months. The induction period for all UC patients in this study did not exceed the standard 2-month duration. Except for 4 patients over 60 who received a dose of 15 mg, 26 patients (86.7%) received a dose of 30 mg of UPA for maintenance therapy.

Disease extent was classified according to the Montreal classification (E1: proctitis; E2: left-sided; E3: extensive/pancolitis) [20]. For this study, patients with proctitis were excluded. To more accurately define disease extent, the Montreal classification E3 group was divided into two subcategories: extensive colitis and pancolitis. To assess disease activity, the partial Mayo score (PMS), as recommended in real-world observational cohort studies, was used [21]. Previous publications have noted that PMS correlates well with the full Mayo Score [21,22]. Clinical response was defined as a reduction of at least 3 points or ≥ 30% from the baseline PMS, as advised by phase trials and most real-world studies. Clinical remission was defined as PMS ≤ 2 with all subscores of one or less. Additionally, patients with a baseline PMS of 3–4 were classified as mild, 5–6 as moderate, and greater than 6 as severe. Each patient’s worst state during the previous 24 or 3 days was used to calculate the rectal bleeding score (RBS). When calculating clinical response, a reduction of at least 1 point in RBS or an RBS of 0 or 1 was considered. Active disease was defined as a C-reactive protein (CRP) level greater than 5 mg/L and/or the presence of clinical, endoscopic, or radiographic findings, or a fecal calprotectin (FCAL) value of 150 µg/g or greater.

### 2.2. Data Collection

Three distinct time points were used to evaluate patient data in this study: baseline, the conclusion of the induction phase, and week 24. Laboratory tests were analyzed using blood samples drawn after an 8 h fast, in accordance with standard outpatient clinic protocols. In the hospital laboratory, glucose, HbA1c, urea, creatinine, liver enzymes (aspartate aminotransferase [AST], alanine aminotransferase [ALT], gamma-glutamyl transferase [GGT], alkaline phosphatase [ALP], total bilirubin), albumin, thyroid hormones (thyroid-stimulating hormone [TSH], free thyroxine [FT4]), calcium, lipid profile (total cholesterol, high-density lipoprotein [HDL], low-density lipoprotein [LDL], triglycerides), ferritin, vitamin B12, folic acid, C-reactive protein (CRP), and complete blood count values were measured using Roche Cobas C501 and Beckman Coulter LH 780 analyzers.

Fecal calprotectin (FCAL) levels were measured with the ELISA method using a commercial kit (Immundiagnostik AG, Stubenwald-Allee 8a, 64625 Bensheim, Germany). The measuring range of this test was 13–840 µg/g.

For patients who discontinued treatment due to a loss of response or other reasons during the induction or maintenance phase, FCAL, CRP, and all other laboratory values were recorded at the time of discontinuation. These data were included in the analysis for the relevant evaluation period (induction or week 24).

Due to the retrospective and real-world nature of this study, centralized or blinded endoscopic and histological assessments, which are standard in prospective clinical trials, were not performed for the evaluation of treatment outcomes. All measures of response and remission were based solely on clinical scores, laboratory parameters (e.g., CRP, fecal calprotectin), and treating physician records.

### 2.3. Statistical Analysis

The statistical analyses were performed using SPSS (Statistical Package for the Social Sciences) version 25.0 (IBM Corp., Armonk, NY, USA), and a *p* value of <0.05 was considered statistically significant. Demographic and laboratory data were summarized as the mean ± standard deviation (SD). The Shapiro–Wilk test was used to determine whether the data had a normal distribution. The Independent Samples *t*-test for normally distributed data and the Mann–Whitney U test for non-normally distributed data were utilized to compare continuous variables between the two independent groups (UST and UPA). Categorical variables were presented as frequency and percentage (%). The chi-square test and, where appropriate, Fisher’s Exact Test were applied to compare proportions between groups. To compare measurements at different time points for the same patient (baseline, induction, and week 24), Repeated Measures ANOVA was used for normally distributed data, and the Friedman test or Wilcoxon signed-rank test was used for non-normally distributed data. Using a mixed-model approach within the Generalized Linear Model, considering the Time × Treatment Interaction, the difference in efficacy between the treatment groups was assessed. This approach allowed for the inclusion of missing data from patients who were lost to follow-up, thereby minimizing bias. The effect size was reported with the odds ratio (OR) and its 95% confidence interval (CI).

## 3. Results

### 3.1. Demographic Data and Baseline Characteristics

A total of 89 patients (UST [*n* = 57], UPA [*n* = 32]) who met the inclusion criteria were included in this study between 1 June 2020 and 28 February 2025. There were no statistically significant differences between the two groups regarding demographic parameters, including age, gender, disease duration, and disease extent. The rates of extra-intestinal manifestations, comorbidities, and ankylosing spondylitis were also similar.

At baseline, almost all patients in both groups had moderate to severe ulcerative colitis, and approximately one-third were still smokers. In the UST and UPA groups, 45.6% and 56.3% of patients, respectively, had prior exposure to more than one biological molecule. No significant difference was found between the groups regarding baseline fecal calprotectin (FCAL) and C-reactive protein (CRP) levels (*p* > 0.05).

The only statistically significant difference between the groups was the rate of concomitant steroid initiation (UST 42.1%, UPA 18.8%, *p* = 0.025). The clinical and demographic features of the patients, along with some laboratory data, are presented in detail in Table 1.

All 89 patients with advanced biological agent experience were included in this study. Nearly all patients in both groups had a history of anti-TNF exposure (98.2% in the UST group and 96.9% in the UPA group). In both groups, the most used prior biological agent was infliximab (UST group: 68.4%, UPA group: 65.6%). Due to the more recent introduction of UPA in our country, no patients in the UST group had prior exposure to this agent.

At the start of treatment, 70.2% of patients in the UST group and 81.3% of patients in the UPA group were using azathioprine. In accordance with safety recommendations, azathioprine was discontinued for patients starting UPA, while it was continued for patients using UST. In our nation or clinic, methotrexate is not a frequently used drug for the treatment of inflammatory bowel diseases. Detailed data regarding the use of more than one molecule are shown in Table 2.

### 3.2. Ustekinumab and Efficacy

Of the 57 patients included in this study, 24 (42.1%) had concomitant steroids initiated with UST therapy. One patient could not use the drug due to an allergic reaction that developed within a week of receiving the subcutaneous UST dose. A total of eight patients were considered primary non-responders to induction therapy (between weeks 11 and 14). A total of 48 patients completed induction therapy, and all 48 patients achieved a clinical response. The clinical response rate among the enrolled patients was 84.2% (48/57), and the clinical remission rate (PMS ≤ 2) was 43.9% (25/57).

At baseline, the FCAL level was above 150 µg/g in all but three patients. At the end of induction therapy, this value dropped below 150 µg/g in 50.9% of the patients (29/57). Similarly, while only 1 patient (1.8%) had a C-reactive protein (CRP) level of ≤5 mg/L at baseline, this level dropped below this threshold in 31 patients (54.4%) at the end of induction therapy. At baseline, all patients had a rectal bleeding score (RBS) of more than 2, but a reduction to 1 or less was observed in 49.1% (28/57) of patients at the end of induction therapy (Table 3 and Table 4).

Of the total of 48 patients who responded to induction therapy, 11 required steroid use during maintenance therapy. Nine of these patients, who had a loss of response, had their treatment discontinued between weeks 20 and 24. Two of these patients were referred for surgery to undergo ileal pouch-anal anastomosis (IPAA) due to multiple-drug resistance.

The treatment persistence rate was 81.3% (39/48) at week 24. Concurrently, the clinical remission rate at week 24 was 58.3% (28/48), and the clinical response rate was 77.1% (37/48).

**Figure 1:**
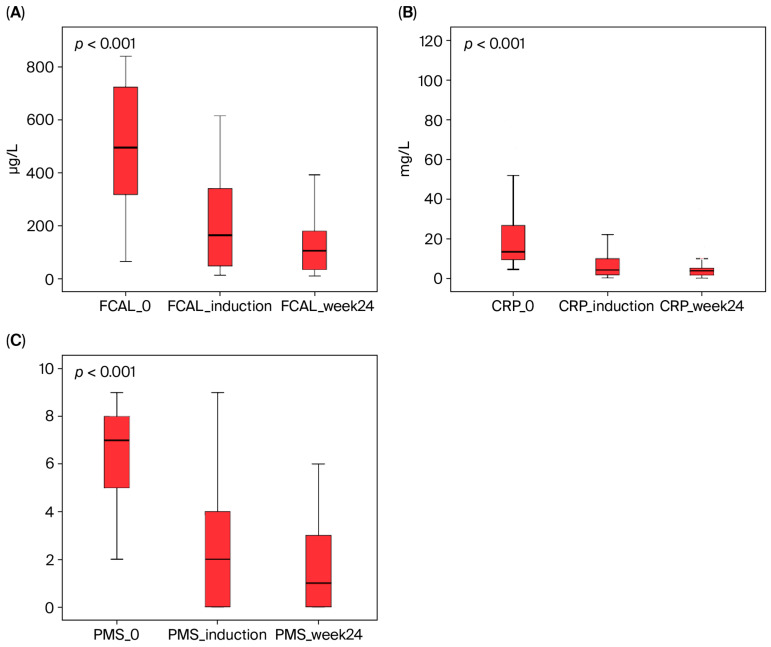
The effect of ustekinumab therapy on fecal calprotectin (FCAL), C-reactive protein (CRP), and partial Mayo score (PMS) in UC patients. These parameters showed a statistically significant decrease from baseline to week 24 (Friedman’s test, *p* < 0.001). (**A**) FCAL, (**B**) CRP, and (**C**) PMS.

At week 24, the proportion of patients with fecal calprotectin (FCAL) levels <150 µg/g was 68.8% (33/48), while the proportion of patients with CRP levels <5 mg/L was 70.8% (34/48). Similarly, the proportion of patients with a rectal bleeding score (RBS) <2 increased to 72.9% (35/48). The steroid-free clinical response rate was 70.8% (34/48) in the maintenance phase (Table 3 and Table 4, Figure 1).

### 3.3. Upadacitinib and Efficacy

Of the 32 patients in the UPA group, one had to stop the medication at week 6 due to symptoms such as shortness of breath and vomiting. Except for 1 patient who was classified as a primary non-responder, 30 patients completed the induction therapy. All these patients achieved a clinical response. The treatment persistence rate and clinical response rate for induction therapy were both 93.75% (30/32). The clinical remission rate (PMS ≤ 2) was 50% (16/32).

At the start of induction therapy, the FCAL level was >150 µg/g in all but one patient. By the end of induction, this value decreased to <150 µg/g in 53.1% of patients (17/32). Similarly, at baseline, all patients had an RBS greater than 1, and this proportion decreased to 40.6% (13/32) at the end of induction therapy (Table 3 and Table 5).

A total of 30 patients who responded to induction therapy continued with maintenance therapy. In this group, four patients developed a secondary loss of response, and their UPA treatment was discontinued between weeks 20 and 23. Another patient’s treatment was stopped due to symptoms such as chest pain, shortness of breath, and tachycardia. During maintenance therapy, six patients were started on steroids.

The proportion of patients who continued treatment until week 24 was 83.3% (25/30). At week 24, the clinical remission rate was 63.3% (19/30), and the clinical response rate was 80% (24/30).

**Figure 2:**
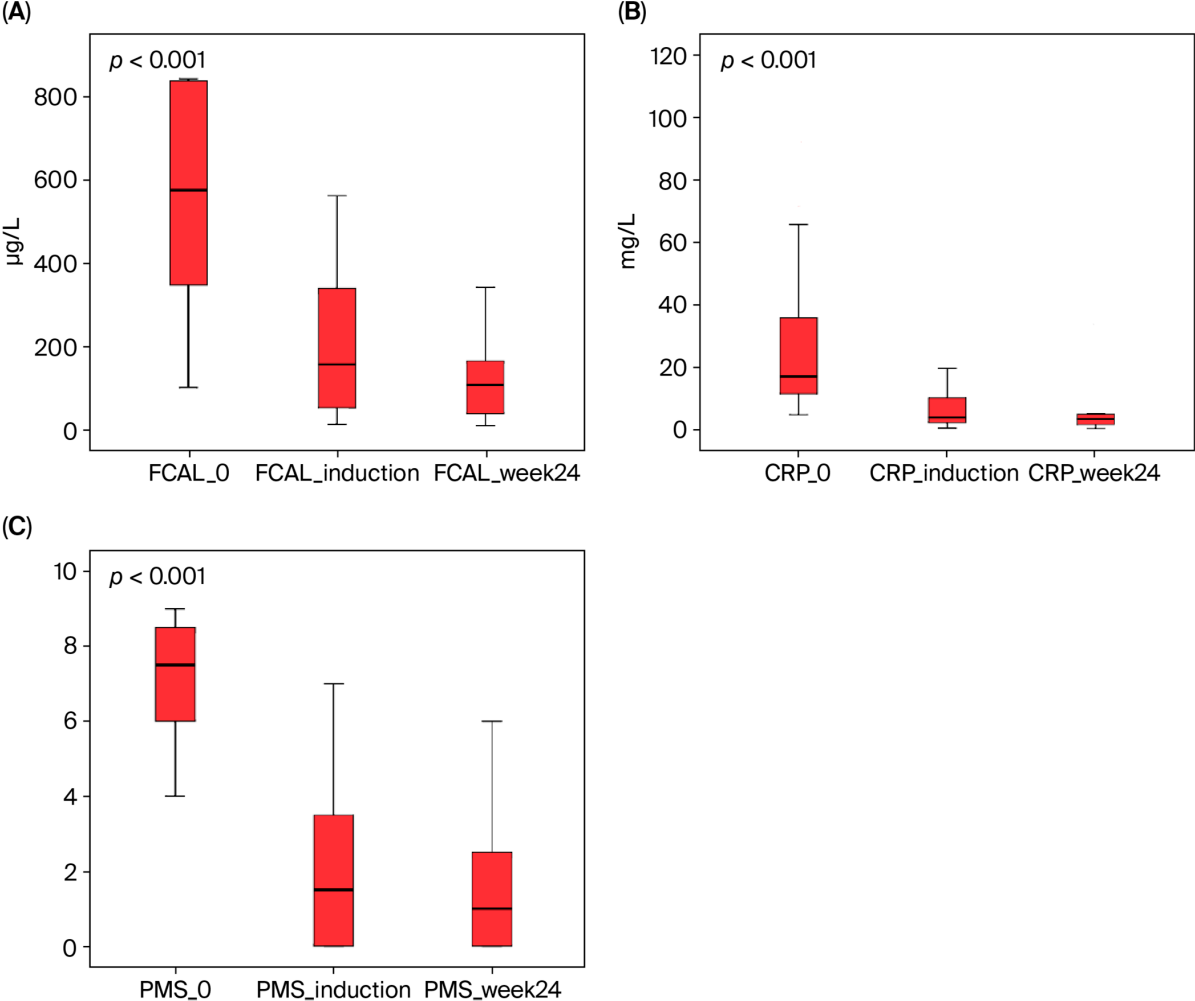
The effect of upadacitinib therapy on fecal calprotectin (FCAL), C-reactive protein (CRP), and partial Mayo score (PMS) in ulcerative colitis patients. These parameters showed a statistically significant decrease from baseline to both induction and week 24 (Friedman test, *p* < 0.001). (**A**) FCAL, (**B**) CRP, and (**C**) PMS.

At week 24, the proportion of patients with FCAL levels <150 µg/g was 70% (21/30), while the proportion of patients with an RBS < 2 was 73.3% (22/30). The steroid-free clinical response rate was 76.6% (23/30) in the maintenance phase (Table 3 and Table 5, Figure 2).

### 3.4. Comparison of Ustekinumab and Upadacitinib Efficacy

In our study, the rates of clinical response, remission, and persistence were compared between patients receiving UST and those receiving UPA during the induction and maintenance (week 24) periods. As shown in Table 3, the clinical response and persistence rates at the end of induction therapy were numerically higher in the UPA group (93.8%) compared with the UST group (84.2%). However, this difference was not statistically significant (*p* = 0.295; OR, 2.81; 95% CI, 0.57–6.87). Similarly, there was no statistically significant difference between the clinical remission rates (*p* = 0.577; OR: 1.28, 95% CI: 0.54–3.05).

**Figure 3:**
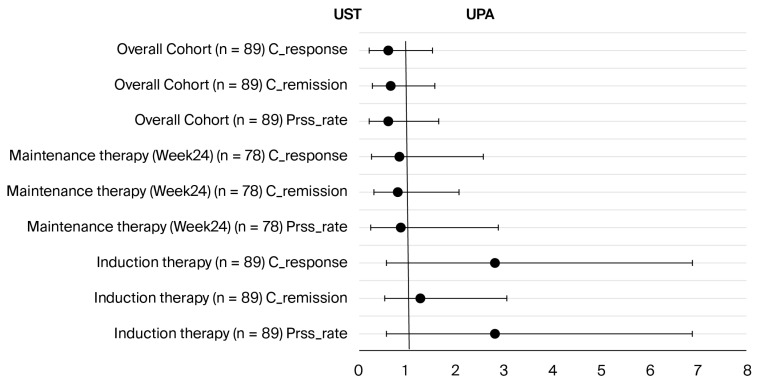
Comparison of odds ratios for clinical response, remission, and persistence rates of ustekinumab and UPA therapies at induction, at week 24, and in the overall cohort. No statistically significant difference was found in any comparison (all *p* > 0.05). Abbreviations: Prss_rate: persistence rate; C_remission: clinical remission; C_response: clinical response; UST: ustekinumab; UPA: upadacitinib.

At the end of maintenance therapy (week 24) and in the overall cohort evaluation, there was no statistically significant difference between the two groups regarding clinical response, remission, and persistence rates (all *p* values were >0.05). Figure 3 supports these findings by showing that the confidence intervals of the odds ratios for all parameters crossed 1.

**Figure 4:**
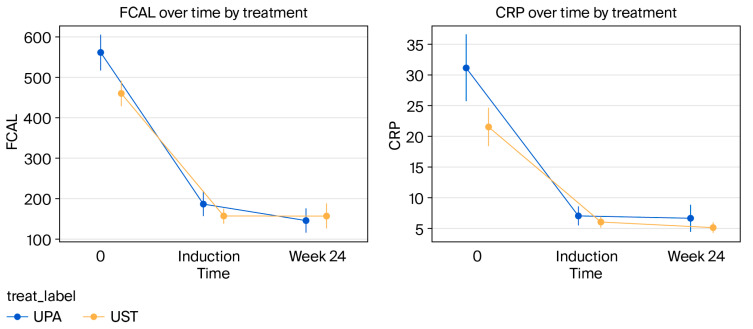
Comparison of the effects of biologic agents on fecal calprotectin (FCAL) and CRP levels. Changes in both fecal calprotectin (FCAL) and CRP levels from baseline to week 24 showed a statistically significant difference between the UST and UPA groups (for FCAL, *p* = 0.018, 95% CI [0.005, 0.031]; for CRP, *p* = 0.026, 95% CI [0.003, 0.048]).

In both treatment groups, fecal calprotectin (FCAL) and C-reactive protein (CRP) values showed a statistically significant decrease from baseline to week 24. Analyses comparing the changes between the groups revealed that the UPA group showed a more effective reduction in FCAL and CRP values than the UST group (*p* = 0.018 and *p* = 0.026, respectively). These findings demonstrate a statistically significant difference in biochemical treatment response between the two groups (Figure 4).

### 3.5. Adverse Events

To evaluate the effect of UST and UPA on laboratory parameters, data from baseline and week 24 were compared. As no abnormal findings were detected in these parameters during interim checks, the analyses and tables include data only from baseline and week 24. Additionally, nine patients from the UST group and two from the UPA group who did not complete the induction phase were excluded from the analysis. No adverse events related to laboratory parameters were observed in these patients during their follow-up period. For patients who discontinued the drug during maintenance therapy due to a loss of response, their laboratory data at the time of discontinuation are included in the week 24 results.

In both treatment groups, no statistically significant changes were detected in liver, renal, and thyroid function tests (AST, ALT, GGT, ALP, total bilirubin, creatinine, and TSH) compared with baseline (all *p* > 0.05). However, statistically significant increases were recorded at week 24 compared with baseline in both the UST group (*p* = 0.002 for albumin; *p* < 0.001 for calcium) and the UPA group (*p* < 0.001 for albumin; *p* = 0.002 for calcium).

Upon examining lipid parameters, the UPA group showed statistically significant increases in total cholesterol (*p* < 0.001), LDL cholesterol (*p* < 0.001), and HDL cholesterol (*p* < 0.001) levels at week 24; however, no significant change was observed in these parameters in the UST group.

In both groups, hematological parameters showed statistically significant decreases from baseline, including platelet counts (*p* < 0.001 and *p* = 0.001), white blood cell (WBC) counts (*p* < 0.001 and *p* < 0.001), neutrophil counts (*p* < 0.001 and *p* = 0.001), and lymphocyte counts (*p* = 0.003 and *p* = 0.014). While a significant increase in hemoglobin levels was detected in the UPA group (*p* = 0.010), no significant change was observed in the UST group (*p* = 0.180). All these findings are summarized in Table 6.

The comparison of adverse event profiles is summarized in Table 7. In both groups, the most frequently observed adverse events were nasopharyngitis, headache, and arthralgia. There was no statistically significant difference in the incidence rates of these events between the groups (*p* > 0.05). The only statistically significant difference observed in this study was in the incidence of acne; the rate in the UPA group (12.5%) was significantly higher than in the UST group (1.8%) (*p* = 0.045). Serious adverse events such as severe infections, malignancies, major cardiac events, or venous thromboembolism, as well as any deaths, were not observed in either group.

The number of patients who discontinued treatment due to adverse events was low in both groups. One patient in the UST group stopped treatment due to an allergic reaction following a subcutaneous injection. In the UPA group, two patients discontinued treatment: one at week 6 due to persistent symptoms of shortness of breath and vomiting, and the other due to continued complaints of chest pain, shortness of breath, and tachycardia, despite the absence of any objective findings

## 4. Discussion

As the number of targeted therapies for UC increases, the clinical positioning, comparative efficacy, and safety profiles of these agents are becoming increasingly important. In this context, UST and UPA stand out as two prominent molecules with different mechanisms of action in the biologically experienced patient cohort who are non-responsive or intolerant to anti-TNF therapy. This study aimed to contribute to the limited data in the literature by comparatively evaluating the efficacy, safety, and laboratory findings of UST and UPA in biologically experienced patients with UC. Specifically, the limited sample size (*n* = 57 vs. *n* = 32) restricts the statistical power to definitively rule out clinically meaningful differences. Therefore, non-significant findings—such as the lack of a statistically significant difference in clinical remission rates between the two groups—should be interpreted with caution. These numerical differences are best viewed as hypothesis-generating for future, adequately powered comparative trials, rather than conclusive evidence of equivalence.

The UNIFI phase trial, which evaluated the efficacy of UST in induction and maintenance therapy, was a major turning point in the treatment of UC [5]. In this study, the biologically experienced patient subgroup achieved clinical response rates of 57.2% at week 8 and clinical remission rates of 12.7%. In our study, the clinical response rate of 84.2% achieved at week 14 during the induction period is even higher than the 77.6% response rate obtained after the week 16 rescue dose in the UNIFI study. This finding suggests that the induction efficacy of UST in real-world practice may be better than that reported in clinical trials. Regarding the maintenance period, while the UNIFI study reported a clinical remission rate of 39.6% at week 44 in biologically experienced patients, our study found a higher clinical remission rate of 49.1% at week 24. This higher remission rate may be attributable to the characteristics of our cohort or the more intensive follow-up of patients in the early stages.

Along with phase trials, real-world data also support the efficacy of UST. In a multicenter study by Molander et al. that followed 221 patients for approximately 14.7 months, clinical remission rates of 49% at week 16 and 68% at week 52 were reported in a group consisting entirely of biologically experienced patients [12]. Our clinical remission rate of 49.1% at week 24 in the UST group is similar to the 16-week remission rate reported in that study. Similarly, the STOCUSTE study also evaluated the long-term real-world efficacy of UST [23]. In that study, steroid-free remission was achieved in 78% of patients at 12 months, whereas this rate was only 13% at 3 months. Our steroid-free clinical response rate of 70.8% is similar to the 12-month data from the STOCUSTE study; however, our early steroid-free remission rate was higher than the STOCUSTE data (concomitant steroids were given to 42.1% of the patients started on UST in our study). This difference might be due to variations in baseline disease severity and extent among the patients.

The effect of UST on biochemical parameters is also supported by the literature, in parallel with clinical outcomes. The UNIFI phase trial reported that mean FCAL and CRP levels showed a significant decrease throughout the treatment period [5]. Our study observed similar findings: the high baseline FCAL and CRP levels in the UST group showed a statistically significant decrease during both the induction and maintenance periods (FCAL/CRP at baseline: 478 µg/g and 21.3 mg/L, respectively; at induction: 201 µg/g and 7.49 mg/L; at week 24: 157 µg/g and 5.14 mg/L). These findings suggest that improvements in laboratory parameters also support clinical response. Finally, a systematic review reported clinical remission rates for the induction phase of UST between 16% and 61%, and for the maintenance phase between 33% and 79% [24]. The findings of our study align with the upper range of this wide spectrum in the literature, confirming that UST is a highly effective treatment option for biologically experienced UC patients in real-world practice.

The efficacy of UPA in moderate to severe UC was demonstrated in the U-ACHIEVE and U-ACCOMPLISH Phase 3 studies [14]. In these trials, clinical remission rates of between 26.1% and 33.5% and clinical response rates of between 72.6% and 74.5% were reported in the induction phase, according to the adapted Mayo score. In our study, the induction phase clinical response rate of 93.75% and clinical remission rate of 50% were higher than those observed in the phase trials. Regarding the maintenance phase, the U-ACHIEVE maintenance study reported a clinical remission rate of 51.7% and a corticosteroid-free remission rate of 68% at week 52. In contrast, our study found a clinical remission rate of 63.3% and a corticosteroid-free clinical response rate of 76.6% at week 24. These higher results may be related to factors such as the use of the partial Mayo score and the shorter 24-week follow-up period in our study. Our findings suggest that UPA may have higher efficacy in real-world practice than in clinical trials.

The data from these phase trials are also supported by real-world evidence. A multicenter retrospective cohort study involving 76 patients with prior exposure to advanced therapies reported a clinical response rate of 84.2% with UPA in the induction phase, which is very similar to the 93.75% response rate observed in our study [15]. In a broader context, a meta-analysis by Taxonera et al., which included 1388 patients, found clinical remission rates of 68.4% at week 8 and 64.6% at weeks 24–36 in biologically experienced patients [25]. These data are similar to our study’s week 24 clinical remission rate of 63.3%. However, we must acknowledge that the vast majority of publications in this meta-analysis consisted of conference abstracts, and they demonstrated significant heterogeneity.

Parallel to the clinical response and remission rates, the effect of UPA on inflammatory biomarkers is also consistent. Phase studies have observed a significant decrease in CRP levels during both the induction and maintenance phases [14]. In our study, the high baseline fecal calprotectin (FCAL) and CRP levels in the UPA group also showed a remarkable decrease during both the induction and maintenance periods. Furthermore, FCAL levels dropped below 150 µg/g in 53.1% of our patients at the end of induction and in 70% at the end of week 24. These biochemical responses show a strong correlation with our clinical outcomes. Regarding treatment persistence, the rates of 93.8% in the induction phase and 83.3% in the maintenance phase in our study are consistent with the rates reported in phase trials and real-world data. The reasons for discontinuation in our two patients who did not complete induction therapy (persistent nausea/vomiting and primary non-response) also parallel the most common reasons for treatment discontinuation reported in the literature.

One of the most important debates in the literature is which of the agents with different mechanisms of action is superior in biologically experienced UC patients. A 2024 evidence synthesis and meta-analysis published by the American Gastroenterological Association (AGA) demonstrated that both UPA and UST have clear superiority in achieving clinical remission compared with placebo in this patient group [26]. In this analysis, UPA was predicted to be the most effective agent for clinical remission in the induction phase, with UST ranking second. Although these findings provide a general idea of the clinical efficacy of the two agents, direct comparisons in real-world practice are of great importance due to the complex treatment histories and heterogeneous patient populations. Our study provides a unique contribution to this debate. The UST and UPA groups were well balanced regarding demographic, clinical, and laboratory characteristics. The only statistically significant difference between the two groups was the rate of concurrent corticosteroid initiation (*p* = 0.025). This difference is likely attributable to UPA’s rapid onset of action, which requires less corticosteroid support, whereas UST’s slower therapeutic effect necessitates more frequent corticosteroid use during the induction phase. While this difference introduces a potential confounding variable (bias) when comparing induction efficacy, our findings regarding steroid use during the subsequent maintenance phase offer important context. Specifically, the rate of new or repeated corticosteroid use due to flares during maintenance treatment was statistically similar between the UST and UPA groups. This suggests that the impact of the initial steroid difference is largely confined to the induction period, lending greater confidence to the comparability of the overall long-term efficacy outcomes. This finding underscores the importance of a drug’s rapid onset of action for effective patient care.

A retrospective cohort study by Dalal et al., which included 218 patients over 16 weeks, is one of the few studies in the literature that directly compared UPA and UST [27]. Even though the UPA group had a higher percentage of previous advanced therapy failures, UPA was statistically significantly better than UST in their study regarding clinical remission and response during the induction phase. Our study presents parallel findings. Although no statistically significant difference was found, higher numerical remission and response rates were achieved with UPA during both the induction phase and the 24-week follow-up. For example, the clinical response rate in the induction phase was 93.75% for UPA versus 84.2% for UST. Similarly, at the end of week 24, remission rates were 59.4% for UPA compared with 49.1% for UST. While studies in the literature that perform this comparison often make indirect comparisons based on the efficacy of phase trials versus placebo, our study is unique in its direct comparison of UPA and UST and its presentation of 24-week data.

Maintaining clinical remission is a critical parameter for successful UC treatment. AGA’s evidence synthesis stated that over one year, UPA (30 mg) showed a numerically higher relative risk (RR = 2.36) for maintaining clinical remission compared with UST; however, this difference was not statistically significant as the confidence interval (95% CI: 0.82–6.76) included 1 [26]. This finding suggests that there is no definitive evidence supporting the superiority of UPA over UST in maintenance therapy. Although the remission maintenance rate was determined to be 68.4% for UST and 78.2% for UPA in our study, this difference did not reach a statistically significant result, similar to the findings in the AGA practice guideline (*p* > 0.05). This indicates that both drugs may have similar long-term efficacy and are reliable options for biologically experienced UC patients.

In parallel with our clinical results, both agents showed similar effects on biomarkers. In both the UPA and UST groups, high baseline FCAL and CRP levels showed a significant decrease during the induction and maintenance periods. Nonetheless, there was a statistically significant difference in the rates of decline in post-treatment biomarker levels between the two groups, with the UPA group experiencing a more marked decline. This finding contradicts the study by Dalal et al., which directly compared the two agents and found similar biochemical remission rates [27]. The results from our study suggest that while both drugs can control inflammation, UPA may modulate this inflammatory response more rapidly and potently.

The limited real-world data directly comparing UPA and UST highlight the need to look at the topic from a broader perspective. Therefore, we turned to studies comparing UST with other JAK inhibitors. The 2025 TORUS real-world study found that Tofacitinib and UST have similar corticosteroid-free symptomatic remission rates in patients with UC who have received prior anti-TNF therapy [28]. However, analyses in subgroups with a history of multiple treatment failures observed that Tofacitinib was more effective. These findings suggest that treatment classes with different mechanisms of action (JAK inhibitors and anti-interleukins) can exhibit similar efficacy profiles, especially in complex patient populations, and that individual patient characteristics play a decisive role in treatment selection.

The results of our study demonstrate that neither UST nor UPA treatment significantly impacted liver, renal, or thyroid function tests during a 24-week follow-up period. This finding is consistent with the results from the UNIFI study and current systematic reviews, which suggest that UST has a low incidence of hepatotoxicity and renal impairment [5,24]. Similarly, rates of liver function abnormalities were reported to be 2.5–10%, and renal function impairment was reported at a rate of 0.1% in UPA’s phase 3 and long-term studies [14,29]. Real-world studies also report fewer negative effects of UPA on liver and renal function tests [16,30], which aligns with our findings. It is believed that the low rates of hepatotoxicity in both our study and other real-world data might be due to the relatively short follow-up periods.

In our study, the observed changes in both groups—including increases in albumin and calcium levels and decreases in platelet and leukocyte counts—are consistent with improved disease activity rather than a direct drug effect. No severe hematological changes, such as the neutrophil count dropping below 500/µL, were observed in our study. In the literature, the rate of neutropenia in patients using UPA during phase trials has been reported to be between 1.2% and 6%, and the rate of lymphopenia between 1.4% and 6%, with most cases being clinically mild [16,29,30]. In studies with UST, the rates of severe hematological side effects were found to be very low [5,24].

In our study, significant increases in total cholesterol, LDL, and HDL levels were detected in the UPA group at week 24, whereas no such change was observed in the UST group. Lipid increases associated with JAK inhibitors have been previously reported [31]. However, large-scale meta-analyses on the clinical implications of these increases have shown that cholesterol elevation linked to UPA use did not lead to an increased risk of cardiovascular events [32]. Therefore, the elevated lipid parameters observed in our study should be monitored, but they are not believed to pose an additional cardiovascular risk in the short term.

In our study, the most frequently observed adverse events in both treatment groups were nasopharyngitis, headache, and arthralgia, and no significant difference in frequency was found between UST and UPA. This result indicates that the safety profiles of UPA and UST are largely similar. However, the incidence of acne was found to be significantly higher in the UPA group (12.5% vs. 1.8%, *p* = 0.045). This finding is consistent with acne development being one of the known pharmacodynamic side effects of JAK inhibitors. During the 24-week follow-up period of our study, no serious adverse events, such as severe infections, malignancies, MACEs, or venous thromboembolism (VTE), or any deaths were observed in either group. This aligns with the UNIFI study’s findings of low rates of serious adverse event rates (3.4% in the induction phase, 8.5% in the maintenance phase) and with systematic reviews reporting low rates of adverse events and serious adverse events for UST (2.6–8.1%) [5,24]. For UPA, published long-term phase studies have reported rates of serious infection (5.1%), herpes zoster (5.4%), MACEs (0.2%), and VTE (0.5%) [29]. The most common adverse events reported in 52-week phase studies for UPA were nasopharyngitis (4–5%) and headache (2–4%) [14]. The low adverse event rates in our study and other real-world studies may be attributed to the short follow-up period and patient selection.

### Study Limitations and Strengths

This study is of a pioneering nature. Despite including all patients within our scope, our primary limitations are the insufficient sample size for a power analysis and the short follow-up period. Additionally, a major shortcoming of our study is the lack of endoscopic and histological evaluation.

To our knowledge, this is the first real-world study directly comparing the efficacy of UPA and UST in a cohort consisting entirely of biologically experienced patients with ulcerative colitis. Previous publications have generally made indirect comparisons using phase or real-world data from a single drug. Such approaches reduce the reliability of findings due to heterogeneity in parameters such as patient selection and evaluation methods between studies. A major advantage of this study is its use of a consistent methodology for both treatment groups. This provides an opportunity for a more direct and reliable comparison of efficacy and safety than previous publications.

## 5. Conclusions

This study demonstrates that both UST and UPA are effective and safe treatment options for biologically experienced ulcerative colitis. Regarding efficacy, while UPA exhibited numerically higher remission and response rates compared with UST, the difference was not statistically significant. A general similarity was also observed in their safety profiles, as neither drug led to a negative effect on liver or renal function. However, increases in total, LDL, and HDL cholesterol levels were detected in the UPA group, whereas UST did not affect these parameters. Our study provides preliminary data for future prospective and more comprehensive studies due to its retrospective design and limited sample size. Ultimately, based on these comparative efficacy and safety data, our findings suggest specific guidance for clinical practice: upadacitinib (UPA) may be the preferred choice for patients requiring a rapid onset of action, while ustekinumab (UST) may be favored for patients with pre-existing or high-risk cardiovascular factors due to its neutral impact on the lipid profile.

## Figures and Tables

**Table 1:** Comparison of demographic, clinical, and select laboratory parameters between groups at baseline.

		Ustekinumab (*n* = 57)	Upadacitinib (*n* = 32)	*p* Value
Gender (male)		30 (52.6%)	17 (53.1%)	0.963
Age (years)	(mean ± SD)	43.3 ± 14.9	42.5 ± 12.2	0.825
Disease duration (years)	(mean ± SD)	7.19 ± 4.18	8.28 ± 4.39	0.439
Location	Pancolitis	15 (26.3%)	9 (28.1%)	0.932
	Extensive	22 (38.6%)	13 (40.6%)	0.963
	Left-sided	20 (35.1%)	10 (31.3%)	0.730
PMS	≤2 (remission)	-	-	
	3–4 (mild)	3 (5.3%)	2 (6.3%)	0.638
	5–7 (moderate)	26 (45.6%)	13 (40.6%)	0.649
	>7 (severe)	28 (49.1%)	17 (53.1%)	0.717
EIM	Enteropathic arthritis	11 (19.3%)	7 (21.8%)	0.762
	Dermatological	4 (7%)	4 (12.5%)	0.347
	Ocular	2 (3.5%)	1 (3.1%)	0.702
	PSC	1 (1.8%)	1 (3.1%)	0.573
Ankylosing spondylitis		5 (8.8%)	3 (9.4%)	0.912
Comorbidities	Diabetes	3 (5.3%)	1 (3.1%)	0.404
	Hypertension	9 (15.8%)	5 (15.6%)	0.941
	CVD	5 (8.8%)	2 (6.3%)	0.720
	Respiratory	3 (5.3%)	2 (6.3%)	0.589
	Renal disease	1 (1.8%)	1 (3.1%)	0.505
	Other	5 (8.8%)	3 (9.4%)	0.941
	Total	26 (45.6%)	14 (43.8%)	0.887
Smoking		20 (35.1%)	10 (31.3%)	0.730
Anti-TNF		56 (98.2%)	31 (96.9%)	0.697
>1 prior biologics		26 (45.6%)	18 (56.3%)	0.345
Concomitant steroid		24 (42.1%)	6 (18.8%)	0.025
Fecal calprotectin (μg/g)	(mean ± SD)	478 ± 243	550 ± 242	0.174
CRP (mg/L)	(mean ± SD)	21.3 ± 23.2	29.9 ± 30.4	0.084

Abbreviations: anti-TNF, anti-tumor necrosis factor; CRP, C-reactive protein; CVD, cardiovascular disease; EIM, extraintestinal manifestation; mean ± std, mean ± standard deviation; PMS, partial Mayo score; PSC, primary sclerosing cholangitis; SD, standard deviation.

**Table 2:** Prior use of biological and immunomodulator agents in the ustekinumab and upadacitinib groups.

	UST (*n* = 57) *n* (%)	UPA (*n* = 32) *n* (%)	*p* Value
Infliximab	39 (68.4%)	21 (65.6%)	0.781
Adalimumab	28 (49.1%)	15 (46.9%)	0.836
Anti-TNF	56 (98.2%)	31 (96.9%)	0.697
Vedolizumab	15 (26.3%)	10 (31.3%)	0.573
Ustekinumab	-	8 (25%)	-
Two biological molecules	23 (40.4%)	14 (43.8%)	0.741
Three biological molecules	3 (5.3%)	3 (9.4%)	0.569
Four biological molecules	-	1 (3.1%)	-
Azathioprine	40 (70.2%)	26 (81.3%)	0.211

Abbreviations: UPA, upadacitinib; UST, ustekinumab.

**Table 3:** Comparison of odds ratios for clinical response, remission, and persistence rates with ustekinumab vs. upadacitinib at induction, week 24, and in overall cohort.

		Ustekinumab	Upadacitinib	*p* Value	OR (95% CI)
Induction therapy (*n* = 89)	Prss_rate	48/57 (84.2%)	30/32 (93.75%)	0.295	2.81 (0.57–6.87)
	C_remission	25/57 (43.9%)	16/32 (50%)	0.577	1.28 (0.54–3.05)
	C_response	48/57 (84.2%)	30/32 (93.75%)	0.295	2.81 (0.57–6.87)
Maintenance therapy (week 24) (*n* = 78)	Prss_rate	39/48 (81.3%)	25/30 (83.3%)	0.650	0.87 (0.26–2.88)
	C_remission	28/48 (58.3%)	19/30 (63.3%)	0.536	0.81 (0.32–2.08)
	C_response	37/48 (77.1%)	24/30 (80%)	0.761	0.84 (0.27–2.57)
Overall cohort (*n* = 89)	Prss_rate	39/57 (68.4%)	25/32 (78.2%)	0.328	0.61 (0.22–1.66)
	C_remission	28/57 (49.1%)	19/32 (59.4%)	0.277	0.66 (0.28–1.58)
	C_response	37/57 (64.9%)	24/32 (75%)	0.325	0.62 (0.23–1.62

Abbreviations: CI, confidence interval; C_remission, clinical remission; C_responce, clinical response; OR, odds ratio; Prss_rate, persistence rate.

**Table 4:** The effect of ustekinumab treatment on clinical and biochemical parameters (PMS, FCAL, CRP, and RBS) in patients with ulcerative colitis.

Ustekinumab				
	Baseline	Induction	24th Week	*p* Value
PMS (mean ± std)	6.12 ± 1.82	2.74 ± 2.62	1.83 ± 2.52	<0.001
PMS ≤ 2 (%, *n*)	0% (0/57)	43.9% (25/57)	58.3% (28/48)	<0.001
FCAL (µg/g) (mean ± std)	478 ± 243	201 ± 180	157 ± 197	<0.001
FCAL < 150 µg/g (%, *n*)	5.3% (3/57)	50.9% (29/57)	68.8% (33/48)	<0.001
CRP (mg/L) (mean ± std)	21.3 ± 23.2	7.49 ± 7.69	5.14 ± 4.86	<0.001
CRP < 5 mg/L (%, *n*)	1.8% (1/57)	54.4% (31/57)	70.8% (34/48)	<0.001
RBS < 2 (%, *n*)	0% (0/57)	50.9% (29/57)	72.9% (35/48)	<0.001

Abbreviations: CRP, C-reactive protein; FCAL, fecal calprotectin; mean ± std, mean ± standard deviation; RBS, rectal bleeding score; PMS, partial Mayo score.

**Table 5:** Efficacy of upadacitinib therapy in ulcerative colitis patients: changes in clinical and biomarker values at baseline, induction, and week 24.

Upadacitinib				
	Baseline	Induction	24th Week	*p* Value
PMS (mean ± std)	7.06 ± 1.63	2.25 ± 2.20	1.77 ± 2.33	<0.001
PMS ≤ 2 (%, *n*)	0% (0/32)	50% (16/32)	63.3% (19/30)	<0.001
FCAL (µg/g) (mean ± std)	550 ± 242	198 ± 167	146 ± 153	<0.001
FCAL < 150 µg/g (%, *n*)	3.1% (1/32)	53.1% (17/32)	70% (21/30)	<0.001
CRP (mg/L) (mean ± std)	29.9 ± 30.4	7.31 ± 8.23	4.98 ± 6.84	<0.001
CRP < 5 mg/L (%, *n*)	6.3% (2/32)	62.5% (20/32)	80% (24/30)	<0.001
RBS < 2 (%, *n*)	0% (0/32)	59.4% (19/32)	73.3% (22/30)	<0.001

Abbreviations: CRP, C-reactive protein; FCAL, fecal calprotectin; mean ± std, mean ± standard deviation; RBS, rectal bleeding score; PMS, partial Mayo score.

**Table 6:** Comparison of laboratory parameters in treatment groups at baseline and at week 24.

	UST (*n* = 48) Baseline (Mean ± std)	UST (*n* = 48) Week 24 (Mean ± std)	*p* Value	UPA (*n* = 30) Baseline (Mean ± std)	UPA (*n* = 30) Week 24 (Mean ± std)	*p* Value
ALT (U/L)	26.2 ± 15.9	25.8 ± 14.9	0.645	21.8 ± 16.3	23.8 ± 15.8	0.087
AST (U/L)	22.2 ± 8.1	22.7 ± 7.7	0.526	21.9 ± 9.1	23.0 ± 8.2	0.270
GGT (U/L)	25.1 ± 17.4	24.9 ± 16	0.869	22.0 ± 10.8	19.6 ± 8.9	0.070
ALP (U/L)	77.4 ± 30.1	75.5 ± 22.1	0.507	76.3 ± 20.3	73.3 ± 21.4	0.154
T.bilirubin (mg/dL)	0.62 ± 0.21	0.63 ± 0.22	0.565	0.60 ± 0.29	0.62 ± 0.25	0.690
Albumin (g/dl)	4.08 ± 0.41	4.26 ± 0.34	0.002	3.96 ± 0.48	4.36 ± 0.37	<0.001
Calcium (mg/dL)	9.21 ± 0.44	9.39 ± 0.48	<0.001	9.11 ± 0.43	9.33 ± 0.52	0.002
Creatinine (mg/dL)	0.77 ± 0.21	0.76 ± 0.23	0.838	0.78 ± 0.25	0.79 ± 0.29	0.671
Glucose (mg/dL)	95.6 ± 19.9	93.8 ± 18.9	0.107	94.5 ± 21.6	93.5 ± 12.3	0.650
HbA1c (%)	5.32 ± 0.61	5.28 ± 0.63	0.889	5.44 ± 078	5.42 ± 0.85	0.954
TSH (µIU/mL)	1.61 ± 0.8	1.65 ± 0.85	0.471	1.64 ± 0.79	1.53 ± 0.72	0.068
Total-C (mg/dL)	176 ± 30	177 ± 31	0.804	178 ± 42	207 ± 50	<0.001
LDL-C (mg/dL)	100 ± 26	101 ± 25	0.862	101 ± 29	120 ± 38	<0.001
HDL-C (mg/dL)	50.8 ± 12.4	50.7 ± 10.8	0.985	50.6 ± 14.5	59.2 ± 14.4	<0.001
LDL-C/HDL ratio	2.1 ± 0.71	2.06 ± 0.62	0.640	2.07 ± 0.68	2.10 ± 0.71	0.594
Triglyceride (mg/dL)	125 ± 56	123 ± 46	0.779	131 ± 71	136 ± 71	0.423
Hgb (g/dL)	13.4 ± 1.45	13.6 ± 1.3	0.180	12.7 ± 1.61	13.2 ± 1.58	0.010
Plt (×10^3^/µL)	342 ± 98	298 ± 82	<0.001	334 ± 97	295 ± 83	0.001
WBC (×/µL)	9921 ± 3307	8100 ± 2436	<0.001	9840 ± 2708	7656 ± 2865	<0.001
Neutrophil (×/µL)	6455 ± 2726	5090 ± 2239	<0.001	6489 ± 2100	4987 ± 2425	<0.001
Lymphocyte (×/µL)	2511 ± 974	2171 ± 681	0.003	2403 ± 1380	1970 ± 864	0.014

Abbreviations: ALT, alanine aminotransferase; AST, aspartate aminotransferase; GGT, gamma-glutamyl transferase; ALP, alkaline phosphatase; mean ± std, mean ± standard deviation; T.bilirubin, total bilirubin; TSH, thyroid-stimulating hormone; Total-C, total cholesterol; LDL-C, low-density lipoprotein cholesterol; HDL-C, high-density lipoprotein cholesterol; triglyceride, triglyceride; Hgb, hemoglobin; Plt, platelet; WBC, white blood cell.

**Table 7:** Comparison of adverse event profiles of ustekinumab and upadacitinib therapies.

Adverse Events	Ustekinumab (*n* = 57) *n* (%)	Upadacatinib (*n* = 32) *n* (%)	*p* Value
Symptoms			
Allergic reactions	1 (1.8%)	0	0.58
Nausea and vomiting	2 (3.5%)	3 (9.4%)	0.25
Headache	2 (3.5%)	2 (6.3%)	0.49
Arthralgia or myalgia	3 (5.3%)	3 (9.4%)	0.44
Infection			
Nasopharyngitis	5 (8.8%)	2 (6.3%)	0.69
Respiratory infections	1 (1.8%)	1 (3.1%)	0.63
Herpes zoster	1 (1.8%)	2 (6.3%)	0.22
Urinary tract infections	1 (1.8%)	1 (3.1%)	0.63
Serious infection	0	0	
Others			
Acne	1 (1.8%)	4 (12.5%)	0.045
Malignancy	0	0	
MACE or VTE or dead	0	0	
Adverse events leading to drug discontinuation	1 (1.8%)	2 (6.3%)	0.22

Abbreviations: MACE, major adverse cardiac events; VTE, venous thromboembolism.

## Data Availability

The datasets generated and analyzed for this study are included in the published article. Further inquiries can be directed to the corresponding author.

## Abbreviations

The following abbreviations are used in this manuscript:

anti-TNF	Anti-tumor necrosis factor
CI	Confidence interval
CRP	C-reactive protein
FCAL	Fecal calprotectin
IBD	Inflammatory bowel disease
IV	Intravenous
JAK	Janus kinase
MACE	Major adverse cardiac event
OR	Odds ratio
PMS	Partial Mayo Score
RBS	Rectal bleeding score
SC	Subcutaneous
UC	Ulcerative colitis
UPA	Upadacitinib
UST	Ustekinumab
VTE	Venous thromboembolism
